# Supplementary material for: A droplet robotic system enabled by electret-induced polarization on droplet
Source: Nat Commun. 2024 Jul 23;15:6220. doi: 10.1038/s41467-024-50520-9 (PMC11266649; doi:10.1038/s41467-024-50520-9)
Supplement: Supplementary file 1 — Supplementary Information [file 41467_2024_50520_MOESM1_ESM.pdf]

Supplementary Information

A Droplet Robotic System Enabled by Electret-induced Polarization on Droplet

Ruotong Zhang[1], Chengzhi Zhang[1,2], Xiaoxue Fan[1], Christina C. K. Au Yeung[1,3], Huiyanchen Li[3], Haisong Lin[1,3], Ho Cheung Shum[1,3*]*

[1]Department of Mechanical Engineering, The University of Hong Kong, Pokfulam Road, Hong Kong, China.
[2]Department of Materials Science and Engineering, Southern University of Science and Technology, Shenzhen, Guangdong, 518055, China.
[3]Advanced Biomedical Instrumentation Centre, Hong Kong Science Park, Shatin, New Territories, Hong Kong, China.

**Corresponding author. Email: linhs@hku.hk (Haisong Lin); ashum@hku.hk (Ho Cheung Shum).*

This file includes:

Supplementary Note 1. Comparison between EPD mechanism and the existing AC/DC-induced liquid polarization.
Supplementary Note 2. Theoretical relationship between maximum velocity and charge density of the electret for EPD-based droplet actuation.
Supplementary Note 3. Theoretical relationship between maximum velocity and droplet volume for EPD-based droplet actuation.
Supplementary Note 4. Comparison of droplet actuation resistance on hydrophilic and hydrophobic surface.
Supplementary Note 5. Theoretical analysis of the attraction between two droplets floating on the oil-air interface.
Supplementary Figure 1. Schematic diagram of the existing common techniques for automated droplet manipulation.
Supplementary Figure 2. Comparison between EPD and EWOD's operable liquid types by demonstrating the actuation of various inorganic/organic liquids.
Supplementary Figure 3. Comparison between EPD and EWOD's compatibility with bio-samples by demonstrating the actuation of various body fluids.
Supplementary Figure 4. Simulation of the actuation of droplet which carries a measured amount of positive charge by positive and negative electrets.
Supplementary Figure 5. Simulation of the droplet's effective actuation distance under electrets with different charge amounts.
Supplementary Figure 6. Optical image of the EPD-based actuation of multiple droplets.
Supplementary Figure 7. Calculation of the force needed for effective actuation.
Supplementary Figure 8. Actuation of human serum, saliva, and urine on oil-based EWOD.
Supplementary Figure 9. The protein adsorption on air-based EWOD.
Supplementary Figure 10. Actuation of protein solution on oil-based EWOD.
Supplementary Figure 11. Comparison of the compatibility with living cells of EPD and oil-based EWOD.

Supplementary Figure 12. EPD-based actuation of droplets containing various samples, including water-in-oil emulsion, oil-in-water emulsion, and particles-in-water.
Supplementary Figure 13. Surface morphology of the superhydrophobic surface.
Supplementary Figure 14. The EPD-based droplet actuation on hydrophilic surface.
Supplementary Figure 15. Adjustment of the maximum actuation velocity by introducing surface surfactant in oil substrate.
Supplementary Figure 16. Comparison of droplet evaporation on the hydrophobic substrate and oil substrate.
Supplementary Figure 17. Simulated electric potential distribution of the EPD gripper.
Supplementary Figure 18. Locations of the four stable equilibrium points generated by EPD gripper according to the simulation result.
Supplementary Figure 19. Simulation of the force generated by slice-shape electret on the droplet at different locations.
Supplementary Figure 20. Comparison of the force generated by slice-shape and gripper-shape electrets on the droplet.
Supplementary Figure 21. Resolution and precision of EPD compared to EWOD and magnetic-based system in experiments.
Supplementary Figure 22. Basic microfluidic functions which can be performed by the EPD-based droplet robotic system.
Supplementary Figure 23. Analysis of droplet merging in EPD-based droplet robotic system.
Supplementary Figure 24. Actuation of human serum, saliva, and urine on air-based EWOD with working voltage of 100 V and 150 V.
Supplementary Figure 25. The standard curve for serum lithium, saliva lithium, and urine lithium established by measuring absorbance of calibration samples at 540 nm and 630 nm.
Supplementary Figure 26. The standard curve for serum lithium, saliva lithium, and urine lithium established by in-situ photography and RGB analysis.
Supplementary Figure 27. The application of the EPD-based droplet robotic system for establishing in vitro cell-bacteria model of inflammation and in-situ detection of inflammatory mediator.
Supplementary Figure 28. EPD force exerted by a downsized EPD gripper.
Supplementary Figure 29. Surface morphology of the microfluidic detection chip through SEM image.
Supplementary Table 1. Comparison between EPD and other common techniques, including EWOD, magnetic, acoustic, and thermal based droplet actuation platform.

Supplementary Note 1. Comparison between EPD mechanism and the existing AC/DC-induced liquid polarization.

The most fundamental physical principle of EPD can be attributed to polarization, a principle that is indeed already present and discussed in the literature about micro-object actuation [1,2]:

$$F_{\text{polarization}} = 4\pi\varepsilon_{\text{m}} r^3 CM(E \cdot \nabla)E$$

where E is the electric field strength, r is the radius of the particle, ε_{m} is the permittivity of the surrounding medium and CM is the Clausius–Mossotti factor related to the effective polarizability of the particle.

The traditional method to polarize liquid is to apply high-frequency and high-voltage AC/DC electric fields in a microfluidic device [3-6]. Despite originating from the same governing principle of polarization as AC/DC-induced polarization, the polarization induced by electrostatic charges deserves to be discussed and explored separately. The equivalent circuit models are different for the AC/DC-induced and electrostatic charges-induced polarization, where the former model is externally connected by a power supply, while the latter model is an isolated system.

In the model of AC/DC electric field [4,7], an external power supply connects the upper and lower electrodes and maintain the voltage U constant. In this closed circuit, both conduction and displacement currents exist and compete frequency-dependently. Thus, CM in the equation above should be expressed as [1,2]:

$$CM = \text{Re}\left[\frac{(\varepsilon_{\text{d}} - j\frac{\sigma_{\text{d}}}{\omega}) - (\varepsilon_{\text{m}} - j\frac{\sigma_{\text{m}}}{\omega})}{(\varepsilon_{\text{d}} - j\frac{\sigma_{\text{d}}}{\omega}) + 2(\varepsilon_{\text{m}} - j\frac{\sigma_{\text{m}}}{\omega})}\right]$$

in which ω is the frequency of the applied electric field, ε and σ are the permittivity and electrical conductivity, and the subscripts d and m represent the droplet and the surrounding media, respectively. Therefore, the AC/DC induced polarization is related to both electrical conductivity and permittivity of the operated droplet, and the frequency setup needs to be customized according to the electrical property of the operated droplet [5,6].

As for the isolated system model of the electrostatic charges, there is no conduction current. CM is thus independent of conductivity and frequency, only related to the permittivity:

$$CM = \frac{\varepsilon_{\text{d}} - \varepsilon_{\text{m}}}{\varepsilon_{\text{d}} + 2\varepsilon_{\text{m}}}$$

Besides the expression of CM, E also differs for two kinds of polarization. In the model of AC/DC electric field, the voltage U is maintained as constant. Meanwhile, the charge Q accumulated on the electrode ($Q = C \cdot U$, supplied by the power source) changes with the overall capacitance C of the system, leading to a variable the electric field strength E [4,7,8]. For example, the equivalent capacitance of the electric double layer at the electrode interface could become very small in some cases [9] (e.g., σ_{m} below 100 mS/m and field frequency below about 15 kHz [10]). At this time, E within the surrounding medium tends to be zero, leading to an undesired shielding effect [10,11].

In contrast, in the equivalent model for polarization induced by electrostatic charges, the charge accumulated on the electret Q is a constant, leading to a stable E:

$$E = \int_{S_{\text{electret}}} \frac{\varphi\, dS}{4\pi\varepsilon_{\text{m}} d^2}$$

in which φ is the surface charge density of the electret and d is the distance from electret.

Based on the analysis above, the polarization induced by AC/DC and electrostatic charges has different impact parameters and limitations due to the difference in their equivalent circuit models.

Supplementary Note 2. Theoretical relationship between maximum velocity and charge density of the electret for EPD-based droplet actuation.

The droplet actuated by EPD should be subjected to two forces, one is the driving force generated by EPD effect, F_{EPD}, and the other is the drag force on the droplet as it moves through the oil layer, f. According to Equation (1) and Stokes Law, we have:

$$F_{\mathrm{EPD}} \propto \varphi^3 \text{ and } f = 6\pi\eta r v$$

where r is the radius of the droplet and is assumed much smaller than the scale of the field nonuniformity[1], η is the viscosity of the oil layer, v is the speed of the droplet.

Therefore, when the droplet accelerates to the maximum velocity, we should have:

$$a = \frac{F_{\mathrm{EPD}} - f}{m} = 0$$

which lead to:

$$F_{\mathrm{EPD}} = f$$

As a result, we can have:

$$v \propto \varphi^2$$

Supplementary Note 3. Theoretical relationship between maximum velocity and droplet volume for EPD-based droplet actuation.

The droplet actuated by EPD should be subjected to two forces, one is the driving force generated by EPD effect, F_{EPD}, and the other is the drag force on the droplet as it moves through the oil layer, f.
According to Equation (1) and Stokes Law, we have:

$$F_{\mathrm{EPD}} \propto V \propto r^3$$

And

$$f = 6\pi\eta r v \propto r$$

where V is the volume of the droplet, r is the radius of the droplet and is assumed much smaller than the scale of the field nonuniformity[1], η is the viscosity of the oil layer, v is the speed of the droplet.
Therefore, the acceleration of the droplet a can be described as:

$$a = \frac{F_{\mathrm{EPD}} - f}{m}$$

where m is the mass of the droplet.
Therefore, when the droplet accelerates to the maximum velocity, we should have:

$$a = 0$$

which leads to:

$$F_{\mathrm{EPD}} = f$$

As a result, we can have:

$$v \propto \frac{r^3}{r} = r^2 \propto V^{2/3}$$

Supplementary Note 4. Comparison of droplet actuation resistance on hydrophilic and hydrophobic surface.

The contact angle hysteresis of droplets on hydrophilic surfaces significantly grows larger than those on hydrophobic surface [12], while the contact area of the droplets also increases [13], thus leading to a substantially larger relative friction force[14,15]:

$$F = w\gamma(\cos\theta_{\mathrm{r}} - \cos\theta_{\mathrm{a}})$$

where w is width of the droplet bottom perpendicular to the direct of the motion, γ is surface tension at liquid-air interface, $\cos\theta_{\mathrm{a}}$ and $\cos\theta_{\mathrm{r}}$ are advancing and receding contact angles of the liquid-solid interface. Compared to a superhydrophobic surface (in the case of $\theta_{\mathrm{a}} = 160°$ and $\theta_{\mathrm{r}} = 150°$)[16], the resistance of a hydrophilic surface (in the case of $\theta_{\mathrm{a}} = 58°$ and $\theta_{\mathrm{r}} = 12°$)[12] can theoretically increase more than 40 times.

Supplementary Note 5. Theoretical analysis of the attraction between two droplets floating on the oil-air interface.

Two droplets of water, which has a lower density than the oil medium, floats at the oil-air interface, will distort the interface. When the deformation of adjacent interfaces of two droplets overlaps, lateral capillary forces are triggered to minimize the net curvature of the interfaces, which leads to the relative motion of the droplets [17]. The lateral capillary forces between two particles of radius R, separated by a distance between their centers L can be roughly described as [18,19]:

$$F = -2\pi\sigma Q_1 Q_2 q K_1(qL)$$

where

$$Q_i = r_i \sin\varphi_i,\ (i = 1,2) \text{ and } q^2 = \Delta\rho g/\sigma$$

Here σ is the surface tension, φ is the meniscus inclination angle, r_1 and r_2 are the radii of the interfacial contact lines, $K_1(x)$ is the first-order modified Bessel function of the second kind, $\Delta\rho$ is the difference in the mass densities of the upper and lower fluids forming the fluid interface and g is the gravity acceleration. $\varphi > 0$ for light droplets, while the same sign of φ will lead to the attractive force.

Under the force of attraction, the two droplets will gradually come closer and drain the trapped oil between them gradually [20,21]. Below a critical value (typically < 100 Å), the surfaces spontaneously confluence at one or several points and form a thin liquid bridge between two droplets, finally resulting in the merging of them [21-23].

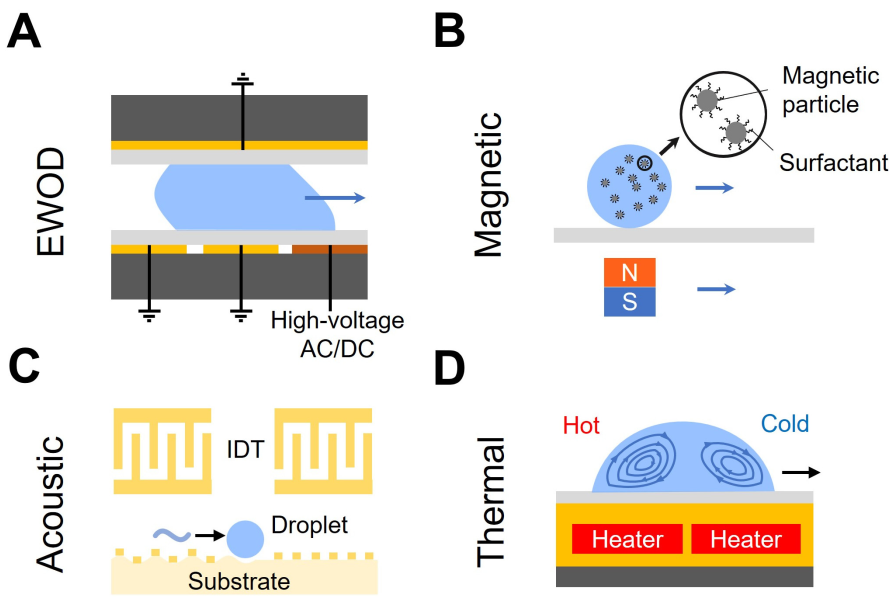

Supplementary Figure 1. Schematic diagram of the existing common techniques for automated droplet manipulation. (**A**) Electrowetting-on-dielectric (EWOD), which actuates droplet by altering the droplet contact angle through electric field. (**B**) Magnetic-based droplet actuation, which achieves magnetic field-based droplet actuation by adding magnetically responsive particles to the droplets. (**C**) Acoustic-based droplet actuation, which actuates droplet by generating surface acoustic wave on piezoelectric substrates. (**D**) Thermal-based droplet actuation, which drives droplet by inducing Marangoni stresses through temperature gradient.

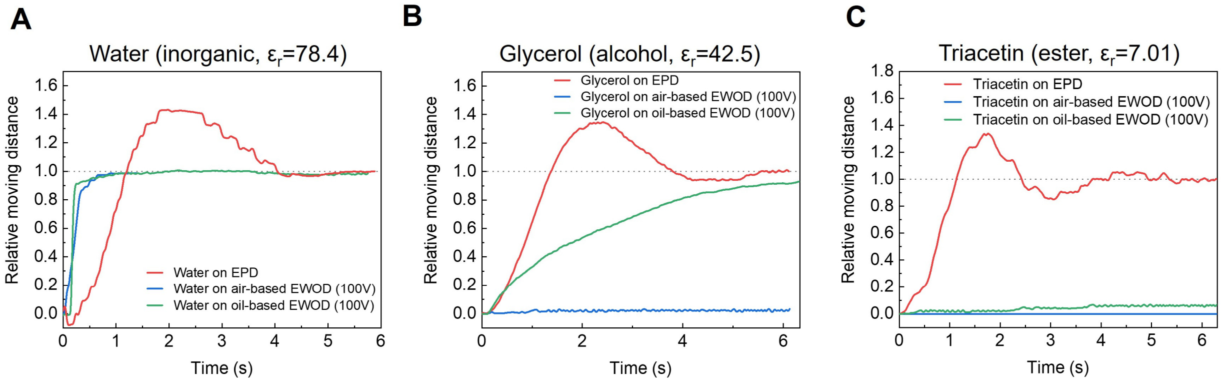

Supplementary Figure 2. Comparison between EPD and EWOD's operable liquid types by demonstrating the actuation of various inorganic/organic liquids. The demonstrated liquid types include (**A**) water (inorganic), (**B**) glycerol (alcohol), and (**C**) triacetin (ester). The position of the front edge of the droplet normalized by the distance between two neighboring coils/electrodes is defined as the relative moving distance. The result indicates that EWOD faces challenges when actuating organic liquids with low dielectric constants, even when oil surrounding is introduced. In contrast, EPD can actuate them smoothly without changing any of the parameter settings, showing EPD's generality of various liquids.

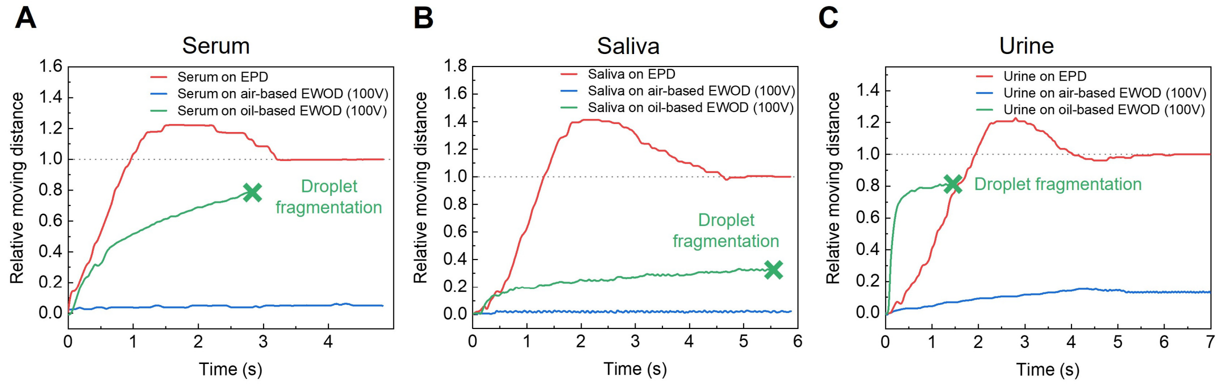

Supplementary Figure 3. Comparison between EPD and EWOD's compatibility with bio-samples by demonstrating the actuation of various body fluids. The demonstrated body fluids include (**A**) serum, (**B**) saliva, and (**C**) urine. The position of the front edge of the droplet normalized by the distance between two neighboring coils/electrodes is defined as the relative moving distance. The result indicates that air-based EWOD faces challenges when actuating biological fluids. When silicone oil environment is introduced to EWOD, the actuation of three body fluids gets improved to varying degrees. However, all three body fluids experience droplet fragmentation during actuation due to the high viscous resistance induced by the oil environment, which prevents subsequent continuous actuation [14]. In contrast, EPD can actuate them smoothly without changing any of the parameter settings, showing EPD's compatibility with body fluids.

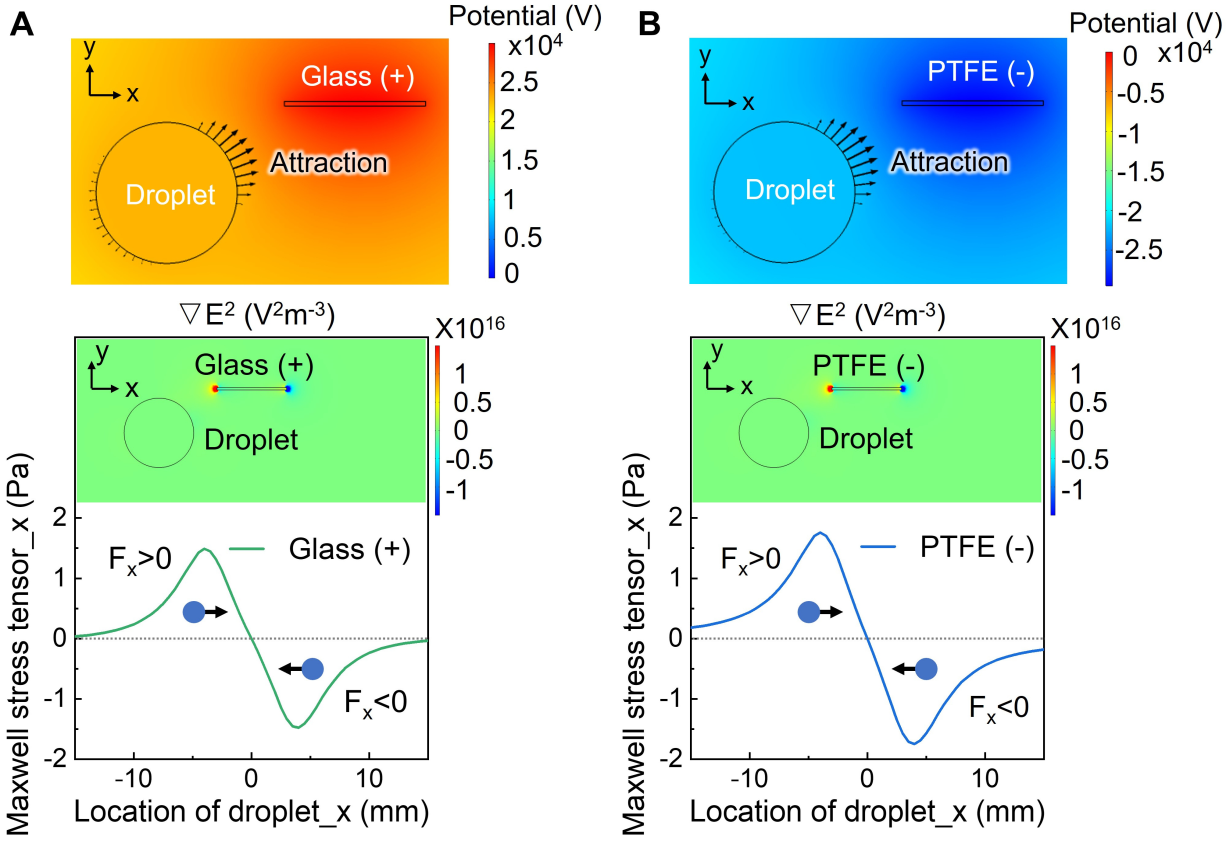

Supplementary Figure 4. Simulation of the actuation of droplet which carries a measured amount of positive charge by positive and negative electrets. (**A**) The simulated ∇E^2 (which should be proportional to the force applied on droplet theoretically) generated by positive electret (glass) and the average Maxwell stress tensor exerted on the droplet carrying a measured amount of positive charge (2×10^{-4} C/m^3). Arrows represent Maxwell stress tensor applied on droplet. Electret is located at 0 mm with the width of 6 mm and height of 0.2mm. The direction of Maxwell stress tensor is the same as the location coordinate. The generated force is directed toward the electret, showing that the droplet is attracted by the positive electret. (**B**) The simulated ∇E^2 (which should be proportional to the force applied on droplet theoretically) generated by negative electret (PTFE) and the average Maxwell stress tensor exerted on the droplet carrying a measured amount of positive charge (2×10^{-4} C/m^3). Arrows represent Maxwell stress tensor applied on droplet. The generated force is directed toward the electret, showing that the droplet is attracted by the negative electret.

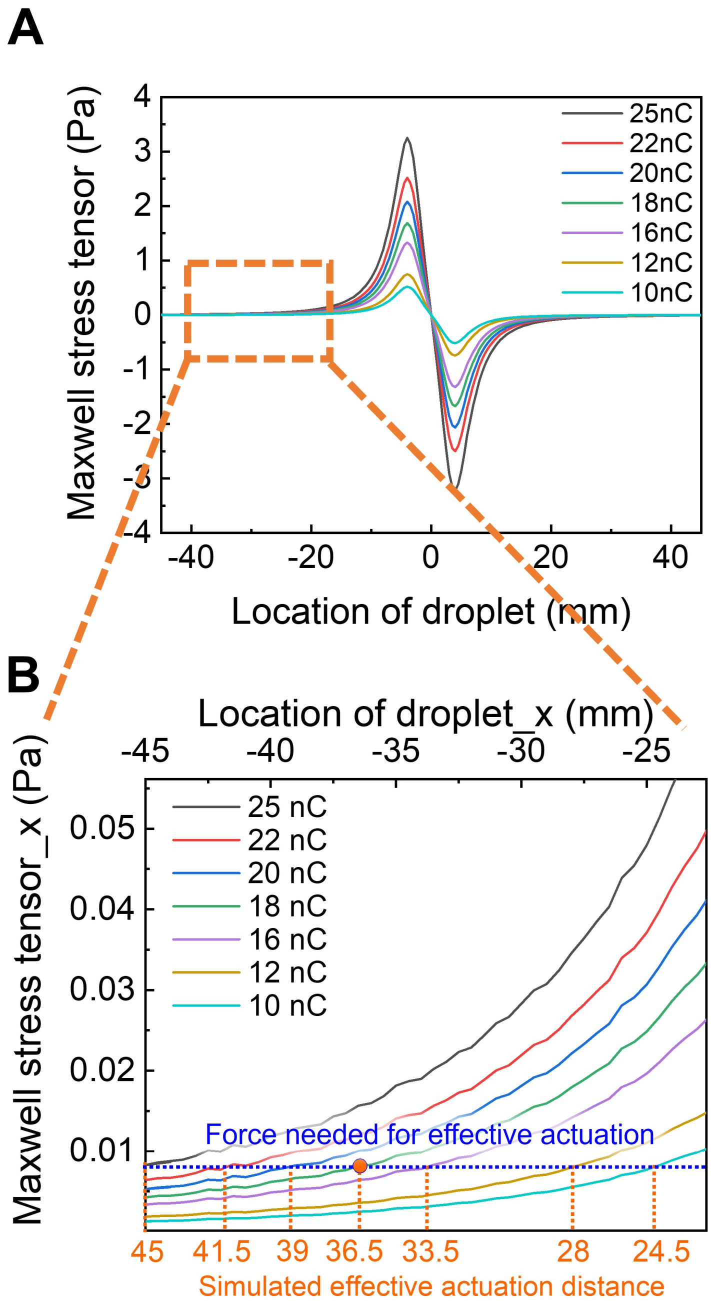

Supplementary Figure 5. Simulation of the droplet's effective actuation distance under electrets with different charge amounts. (**A**) The simulated average Maxwell stress tensor applied on droplet by electrets with different charge amounts. (**B**) Calculation of the effective actuation distance. Droplet's effective actuation distance with electret carrying charge amount of 18 nC is firstly measured (orange point). Then, based on the crossing of the measured effective actuation distance and the simulated Maxwell stress tensor generated by electret with 18 nC, the force needed for effective actuation (dash line) is determined. Based on the crossing of the force needed for effective actuation (dash line) and the simulated force result (solid lines), the simulated effective actuation distance under electrets with other charge amounts can be calculated.

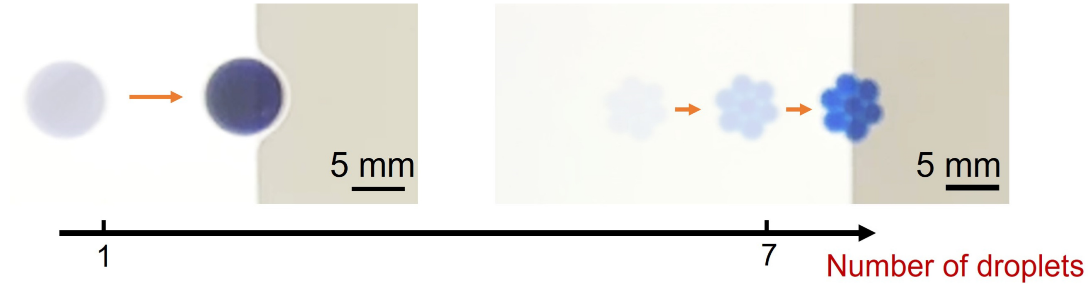

Supplementary Figure 6. Optical image of the EPD-based actuation of multiple droplets. 1 droplet and 7 droplets can both be actuated by EPD, while surface surfactant is introduced in the oil substrate when actuating multiple droplets to prevent droplets from merging.

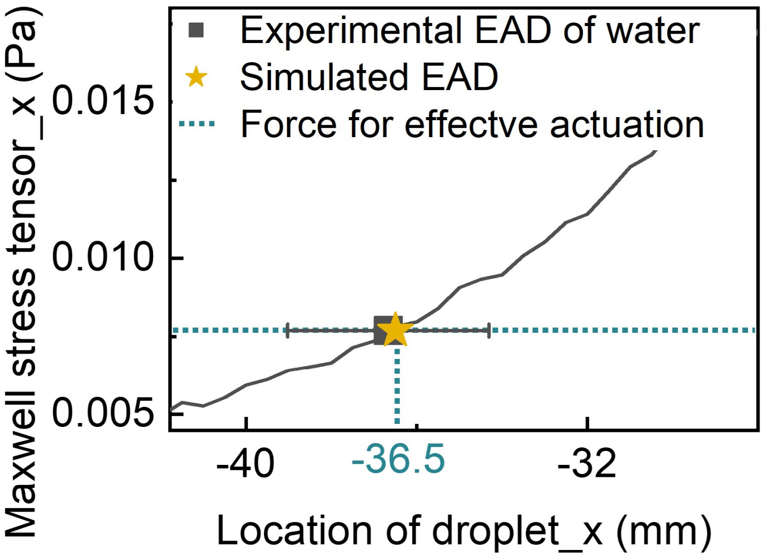

Supplementary Figure 7. Calculation of the force needed for effective actuation. Water droplet's effective actuation distance (EAD) is firstly measured (black square). Then, based on the crossing of the measured EAD of water and the simulated Maxwell stress tensor applied on water droplet, the force needed for effective actuation (dash line) is determined. Error bars, SD.

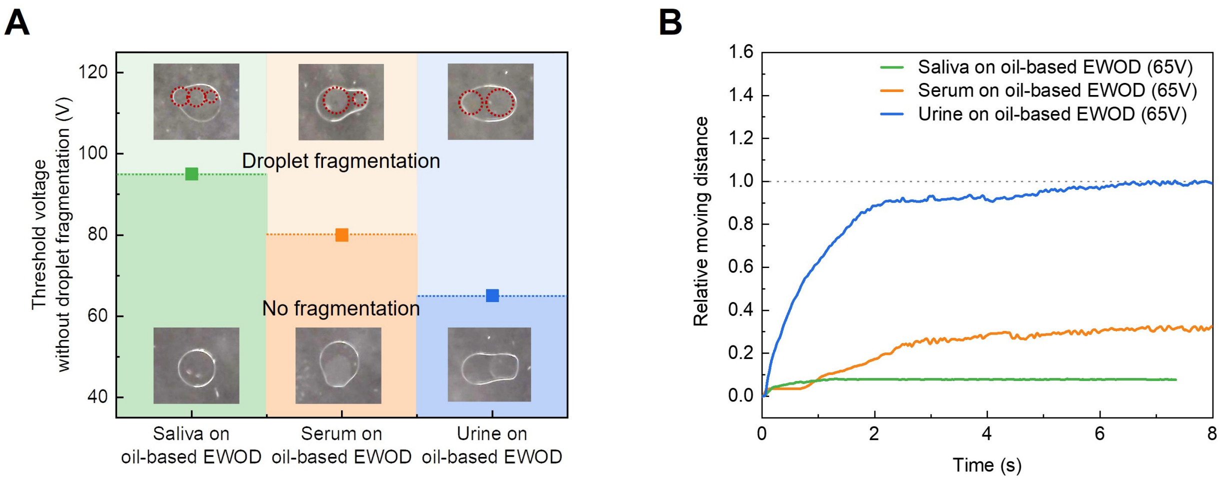

Supplementary Figure 8. Actuation of human serum, saliva, and urine on oil-based EWOD. (**A**) Maximum actuation voltages of serum, saliva, and urine on oil-based EWOD without droplet fragmentation. The result shows that the critical voltages of the three body fluids vary; therefore, the setting of actuation voltage needs to be customized according to the type of body fluids. (**B**) Actuation of human serum, saliva, and urine on oil-based EWOD with a working voltage of 65 V (highest voltage preventing all three body fluids from fragmentation). The position of the front edge of the droplet normalized by the distance between two neighboring electrodes is defined as the relative moving distance. The result demonstrates that the tested oil-based EWOD still faces challenges when actuating body fluids with a higher concentration of protein, while the actuation performance varies significantly among different biofluids.

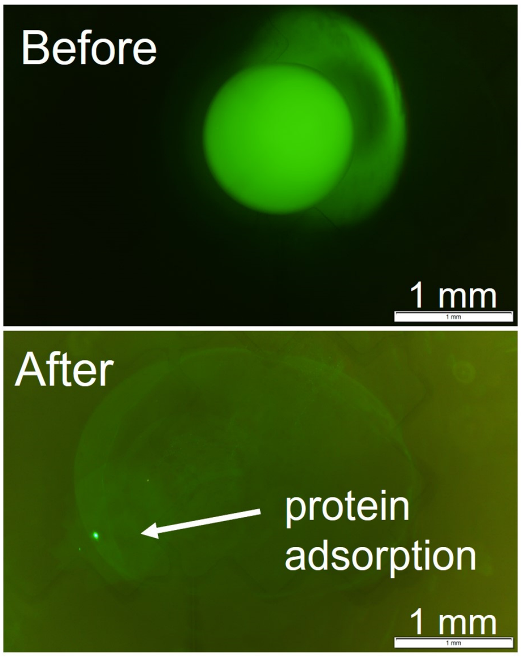

Supplementary Figure 9. The protein adsorption on air-based EWOD. Fluorescence microscope images showing the protein adsorption on double-plate EWOD after actuating a droplet of 0.05 mg/mL FITC-BSA solution, indicated by the change of fluorescence intensity on the substrate.

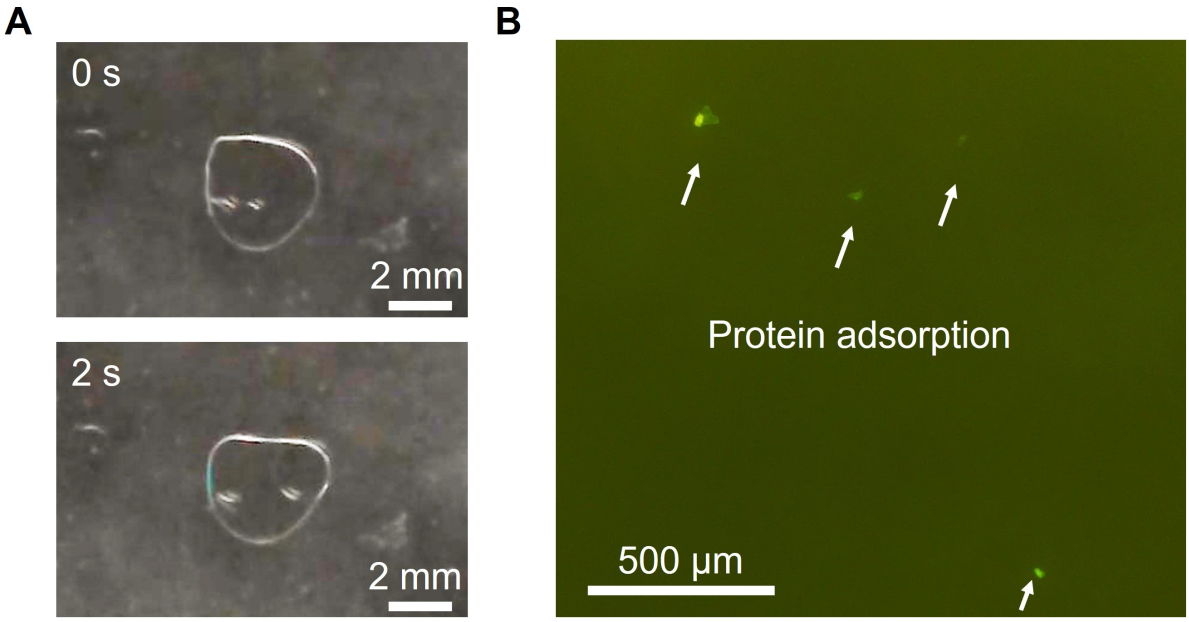

Supplementary Figure 10. Actuation of protein solution on oil-based EWOD. (**A**) Actuation of 0.05 mg/mL FITC-BSA on oil-based EWOD with working voltage of 100 V, in which droplet fragmentation happens and prevents subsequent continuous actuation. (**B**) Fluorescence microscope images showing the protein adsorption on oil-based EWOD after actuating a droplet of 0.05 mg/mL FITC-BSA solution, indicated by the change of fluorescence intensity on the substrate.

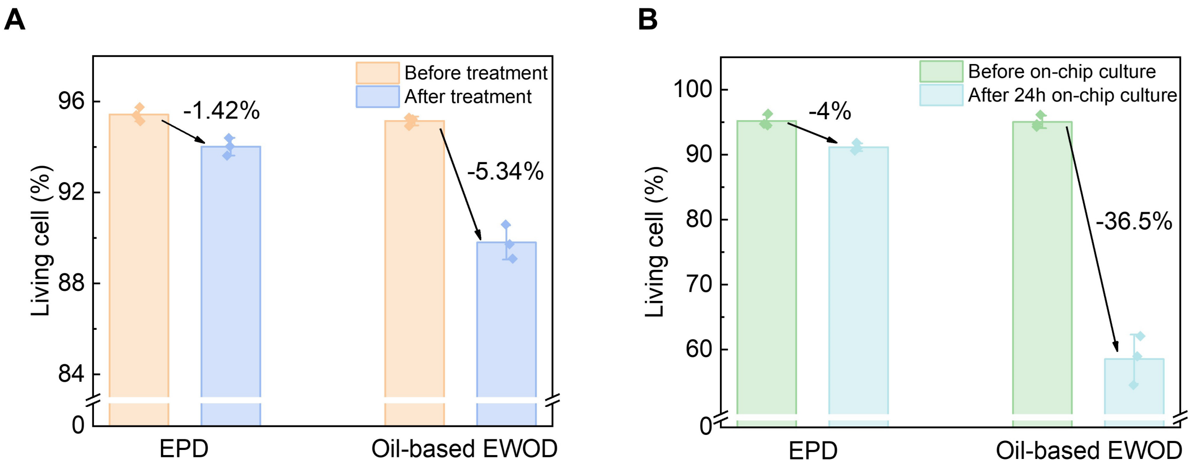

Supplementary Figure 11. Comparison of the compatibility with living cells of EPD and oil-based EWOD. (**A**) Impact of EPD effect and electrowetting effect on cell activity, indicated by the variation of the percentage of living cells after being treated by EPD/ oil-based EWOD for 30 mins and then incubated for 12 hours. The activity of cells treated by oil-based EWOD decreases with a higher degree than that of cells treated by EPD, suggesting that the introduction of oil environment still could not completely avoid the effect of Joule heating and high electric field strength on cell activity. Error bars, SD (n = 3). (**B**) Impact of EPD and oil-based EWOD system setup on cell culture, indicated by the variation of the percentage of living cells after being cultured for 24 hours on EPD/oil-based EWOD without power supply. The percentage of living cells cultured on the oil-based EWOD (58.5%) is significantly lower than that of cells cultured on the EPD (91.2%). This is likely because the silicone oil environment on the double-plate EWOD limits the gas exchange between the droplet and the atmosphere, thus leading to asphyxiation of the cells within the droplet. Error bars, SD (n = 3).

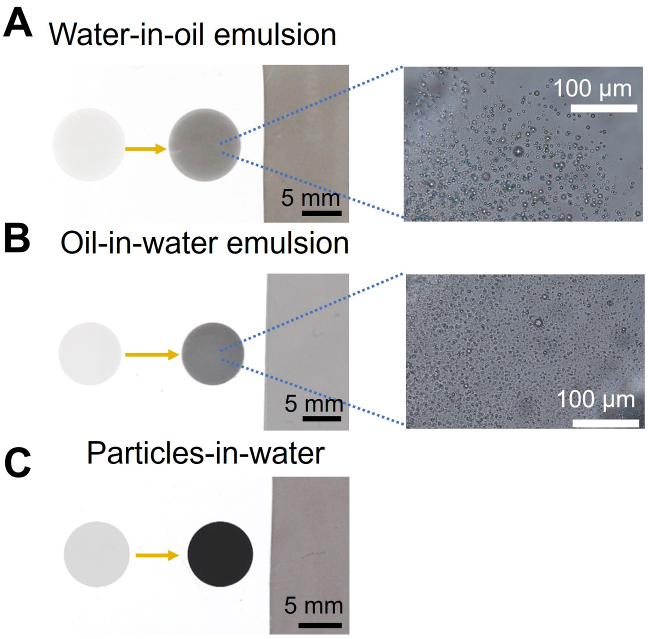

Supplementary Figure 12. EPD-based actuation of droplets containing various samples, including water-in-oil emulsion, oil-in-water emulsion, and particles-in-water. (**A**) Optical images showing EPD-based actuation of 10% water-in-oil emulsion, while the optical image on the right side of this shows the inside of the emulsion. (**B**) Optical images showing EPD-based actuation of 10% oil-in-water emulsion, while the optical image on the right side of this shows the inside of the emulsion. (**C**) Optical images showing EPD-based actuation of particles-in-water (iron oxide magnetic nanoparticles, 30 nm).

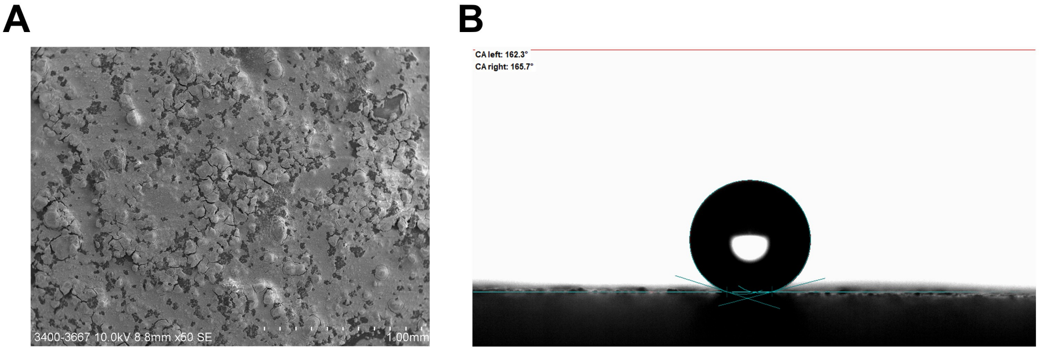

Supplementary Figure 13. Surface morphology of the superhydrophobic surface. (**A**) SEM image of the superhydrophobic surface. (**B**) Measurement of the contact angle of the superhydrophobic surface.

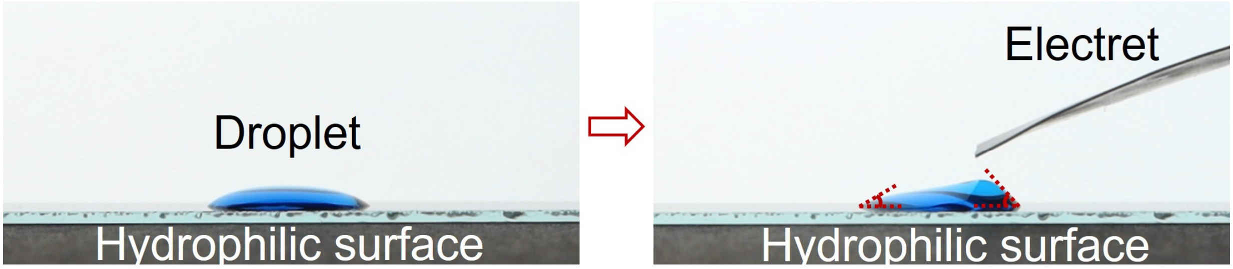

Supplementary Figure 14. The EPD-based droplet actuation on hydrophilic surface. The droplet exhibits a tendency to move with different contact angles on advancing and receding sides. Compared with the hydrophobic surface, the increasing resistance on the hydrophilic surface leads to the failure in droplet actuation.

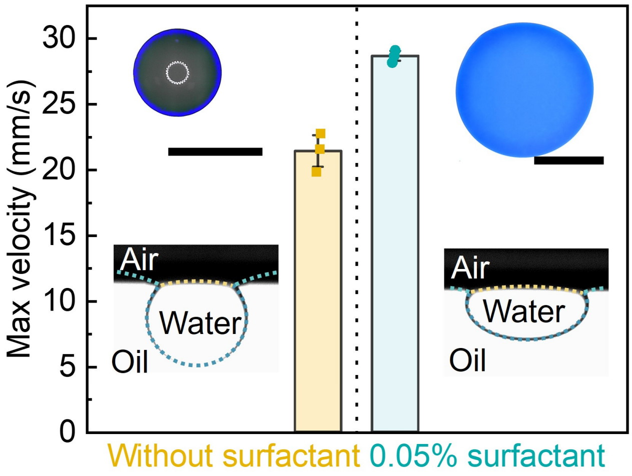

Supplementary Figure 15. Adjustment of the maximum actuation velocity by introducing surface surfactant in oil substrate. Error bars, SD (n = 3).

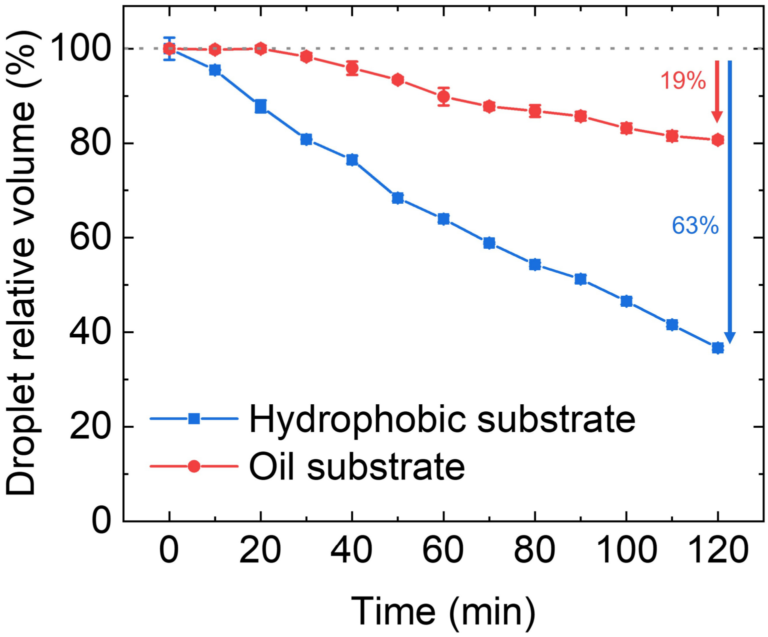

Supplementary Figure 16. Comparison of droplet evaporation on the hydrophobic substrate and oil substrate. Water droplets of 15 μL are dropped on a superhydrophobic substrate with contact angle of 163° and an HFE oil surface, which are placed on an open benchtop. Room temperature and humidity levels are kept constant during the test period. A camera photographs the droplets from above at 10-minute intervals. The dimensions of the droplets are analyzed using ImageJ and the volume change of the droplets is calculated. The results show that droplet evaporation on the oil substrate (red line) is only 30% of that on the hydrophobic substrate (blue line) in two hours at room temperature. Error bars, SD.

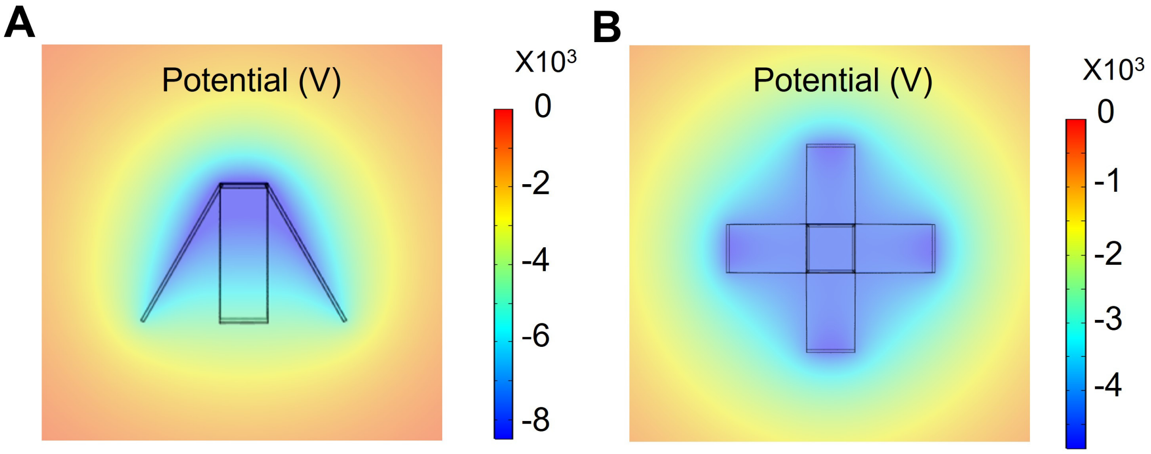

Supplementary Figure 17. Simulated electric potential distribution of the EPD gripper. (A) Side view of the simulated electric potential distribution. **(B)** Bottom view of simulated electric potential distribution.

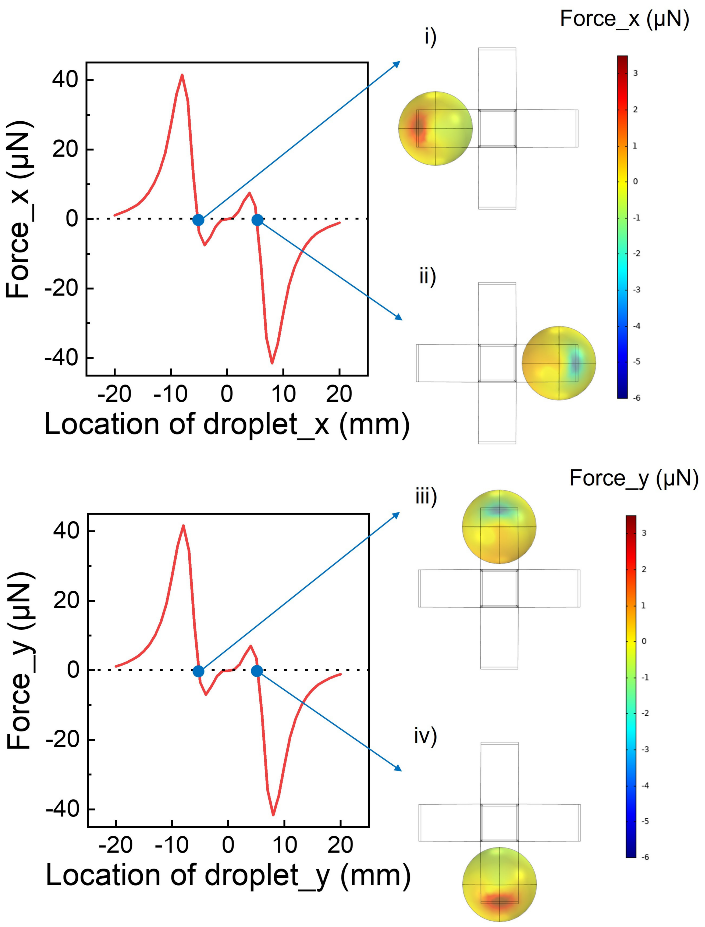

Supplementary Figure 18. Locations of the four stable equilibrium points generated by EPD gripper according to the simulation result.

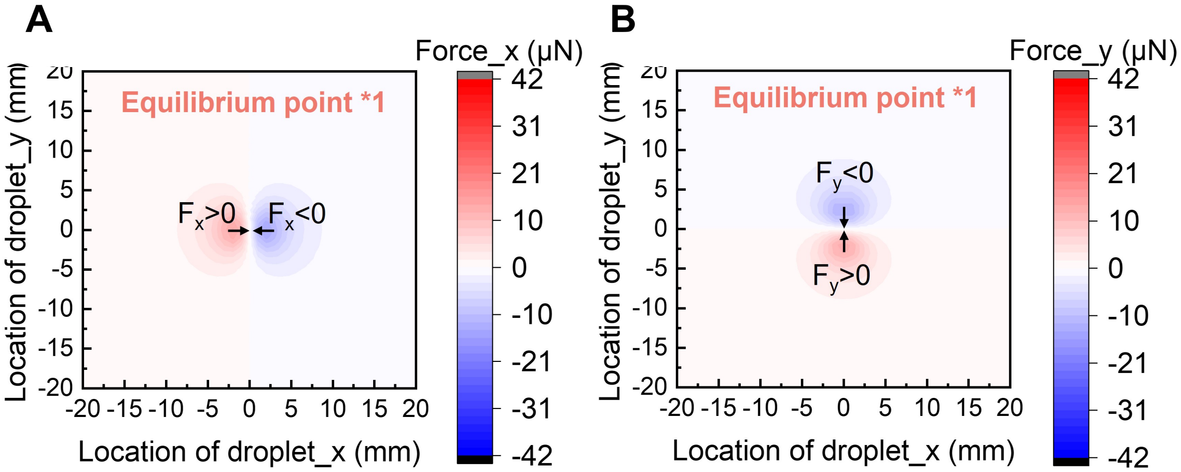

Supplementary Figure 19. Simulation of the force generated by slice-shape electret on the droplet at different locations. (**A**) X component of the force exerted on the droplet at different locations, with one force stable equilibrium points labeled with arrows. The direction of the force in x direction is consistent with the direction of the x coordinate. (**B**) Y component of the force exerted on the droplet at different locations, with one force stable equilibrium points labeled with arrows. The direction of the force in y direction is consistent with the direction of the y coordinate.

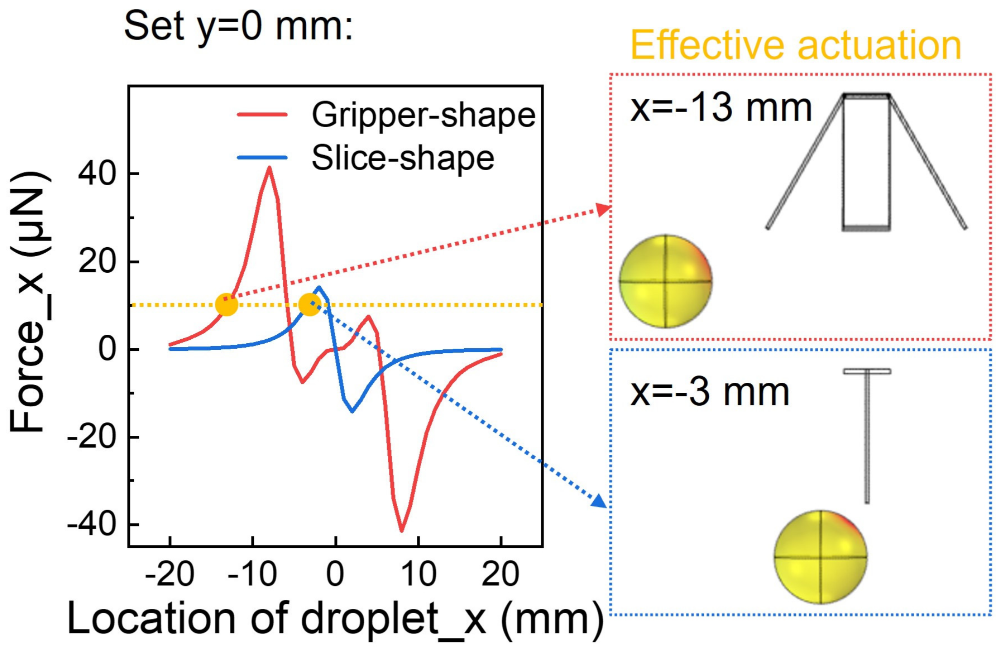

Supplementary Figure 20. Comparison of the force generated by slice-shape and gripper-shape electrets on the droplet. The y coordinate of the droplet is set as 0 mm, and the x component of the force exerted on the droplet is simulated and compared. The yellow dashed line represents the assumed minimum force required for effective actuation [24]. Therefore, the intersection of the dashed line and the force calculated by the simulation (solid line) represents the effective actuation distance (yellow dots). The effective actuation distance of the gripper-shape is proved to be larger than that of the slice-shape electret, which means that the gripper-shape electret can actuate droplet at a longer distance, enhancing the ability of self-assembly and capturing/merging sub droplets. Figures on the right exhibit the force exerted on the droplet at the effective actuation distance by gripper-shape and slice-shape electret, respectively.

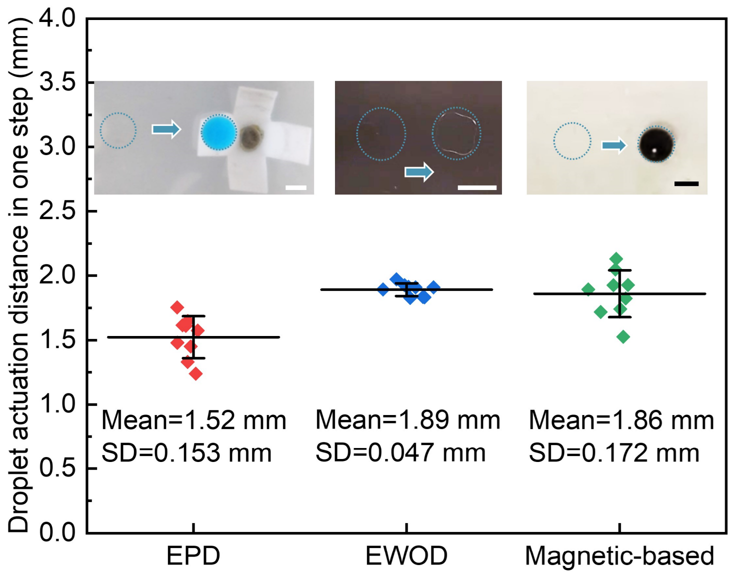

Supplementary Figure 21. Resolution and precision of EPD compared to EWOD and magnetic-based system in experiments. Resolution is defined as the actuation distance in one step, which depends on the coil/electrode/electromagnet size of the platform. In experiments, the resolutions attainable by the three systems are essentially comparable. As for the precision, it is negatively correlated with the standard deviation of actuation distance per step. The results indicate that EWOD has the highest precision, followed by the EPD and magnetic-based systems. Error bars, SD (n = 9). Scale bars: 2 mm.

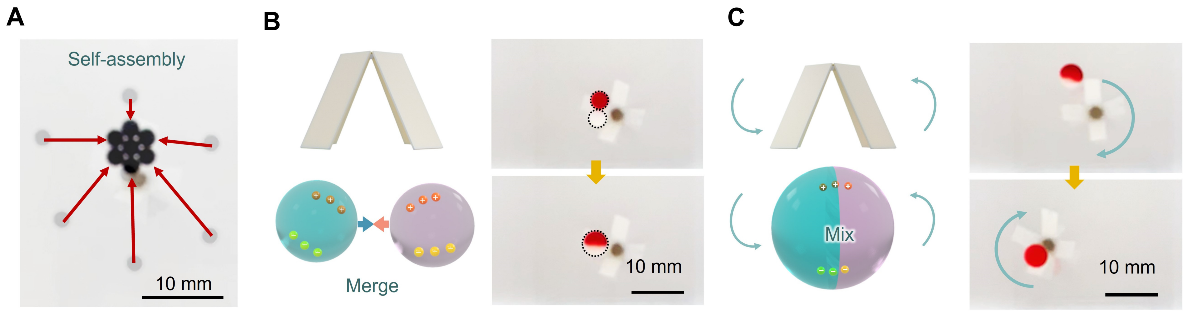

Supplementary Figure 22. Basic microfluidic functions which can be performed by the EPD-based droplet robotic system. (**A**) Self-assembly of multiple droplets. (**B**) Merging of two droplets. (**C**) Mixing of the merged droplets by cyclic motion.

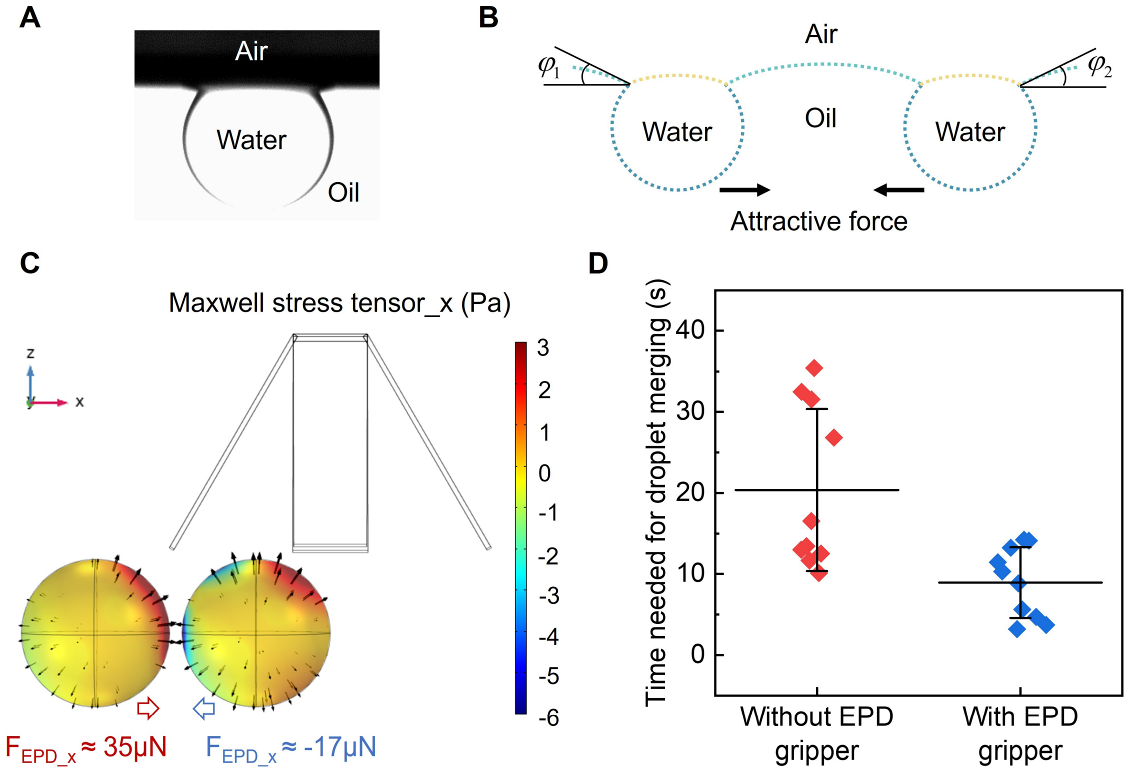

Supplementary Figure 23. Analysis of droplet merging in EPD-based droplet robotic system. (A) The lateral optical image of the floating water droplet at the oil-air interface. (**B**) Schematic diagram of two floating water droplets at the oil-air interface, showing the distorted interface will lead to the attractive force. (**C**) Simulated EPD force applied on two droplets by EPD gripper, indicating that both droplets are subjected to EPD forces pointing at each other. (**D**) Time required for two 20 µL droplets merging, indicating the presence of EPD gripper can help to promote droplet merging. Error bars, SD.

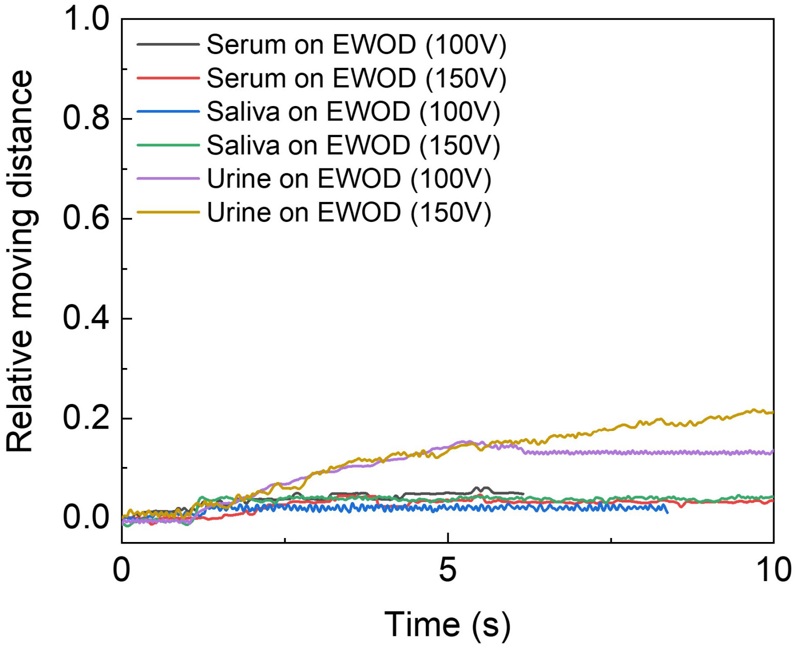

Supplementary Figure 24. Actuation of human serum, saliva, and urine on EWOD with working voltage of 100 V and 150 V. The relative moving distance in y-axis is the relative position of the front edge of the droplet, normalized by the distance between two neighboring coils. The result demonstrates that the tested EWOD faces challenges when actuating body fluids, while the performance improvement brought by the increase of the operating voltage also vary significantly among different biofluids.

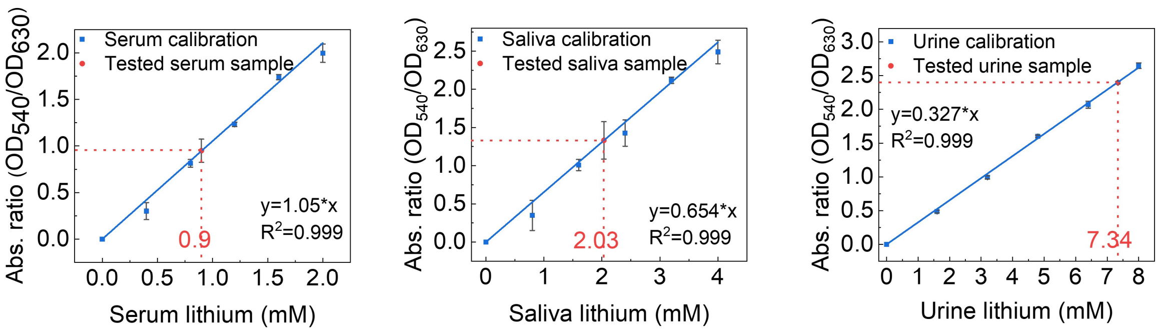

Supplementary Figure 25. The standard curve for serum lithium, saliva lithium, and urine lithium established by measuring absorbance (Abs.) of calibration samples at 540nm and 630nm. Lithium concentration in the tested real sample can be accordingly calculated from it (red points). Error bars, SD (n = 3).

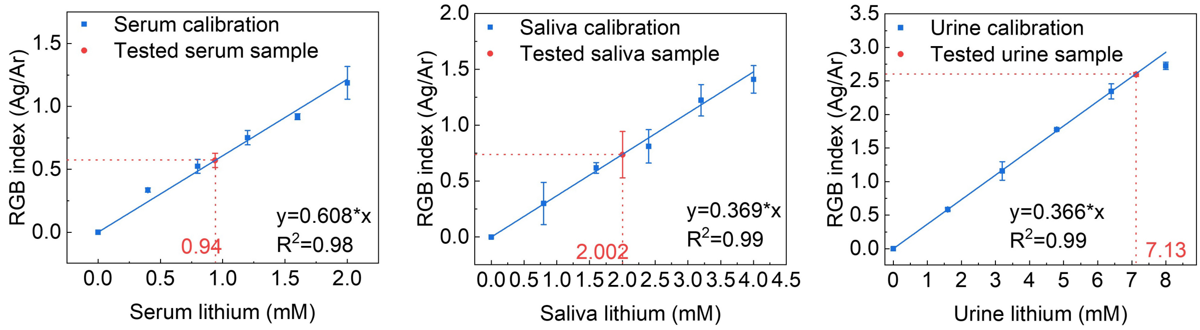

Supplementary Figure 26. The standard curve for serum lithium, saliva lithium, and urine lithium established by in-situ photography and RGB analysis. To establish a standard curve, we subtract the RGB index of the reagent blank (0 μM) from the measured RGB index of other calibration samples. Then we plot the background subtracted RGB index of all calibration samples and calculate the slope of the linear fitted standard curve. A white LED shadowless light acts as a background and a camera (PowerShot G7X Mark III, Canon) is used to take pictures of the mixed droplets. The photographs taken are processed in Adobe Photoshop to obtain the value of R and G though RGB analysis. We define RGB index=Ag/Ar, where Ar=-log(R/255) and Ag=-log(G/255). Lithium concentration in the tested real sample can be accordingly calculated from it (red points). Error bars, SD (n = 3).

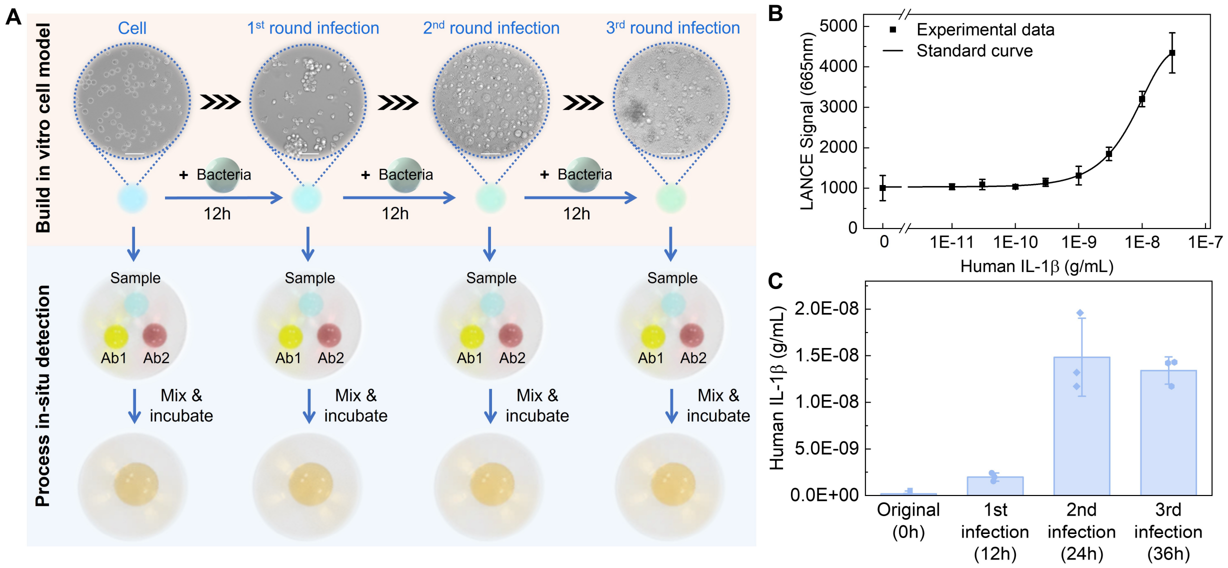

Supplementary Figure 27. The application of the EPD-based droplet robotic system for establishing in vitro cell-bacteria model of inflammation and in-situ detecting inflammatory mediator of human IL-1β. (**A**) Schematic diagram of the workflow of establishing cell-bacteria model of inflammation and in-situ detecting the concentration of inflammatory mediator on EPD-based system. Scale bars: 50μm. (**B**) Standard curve of the concentration of human IL-1β. (**C**) The detected concentration of human IL-1β after 1st, 2nd, and 3rd infection of bacteria. The result shows that the infection of 10% concentration of bacteria can increase the concentration of generated IL-1β by 11 times in 12 hours. The second infection with the same concentration of bacteria can further increase the IL-1β concentration by about 7 times in another 12h. At this point, the cell membrane begins to become transparent, but most cells remain intact. The subsequent third bacterial infection causes most of the cells to break up and die, while the concentration of inflammatory mediators basically remains the same as in the second infection. Error bars, SD (n = 3).

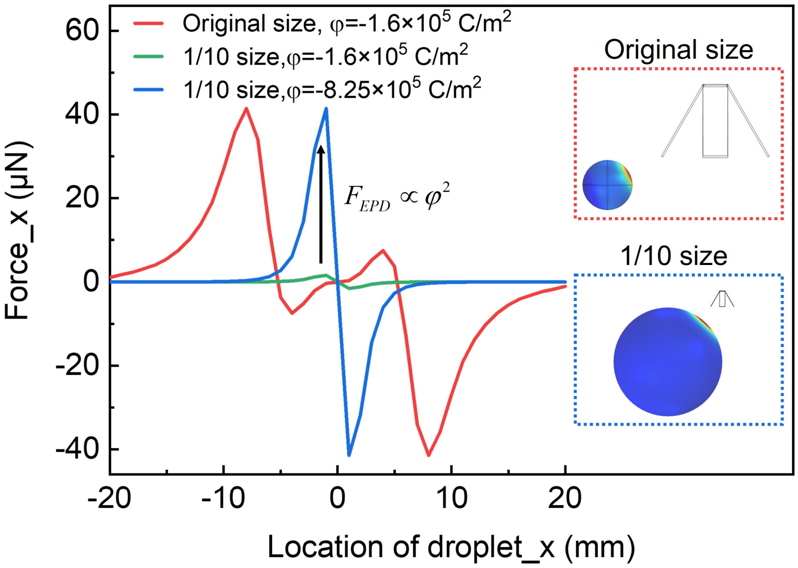

Supplementary Figure 28. EPD force exerted by a downsized EPD gripper. If the EPD gripper (12×12×8 mm) is downsized by 10 times to about 1.2×1.2×0.8 mm, the fabrication of the electret materials is still achievable in the micromachining process. Based on the simulation result, the EPD force exerted by the smaller gripper on the same-volume droplet is also reduced, where the maximum value of the EPD force is reduced by about 26.6 times. To generate a EPD force comparable to the original one, the charge density of the gripper, φ, needs to be increased to about -8.25×10^{-5} C/m^2. The y-coordinate of the gripper is set as 0 mm, and the component of the Maxwell force in the x-direction is calculated and compared.

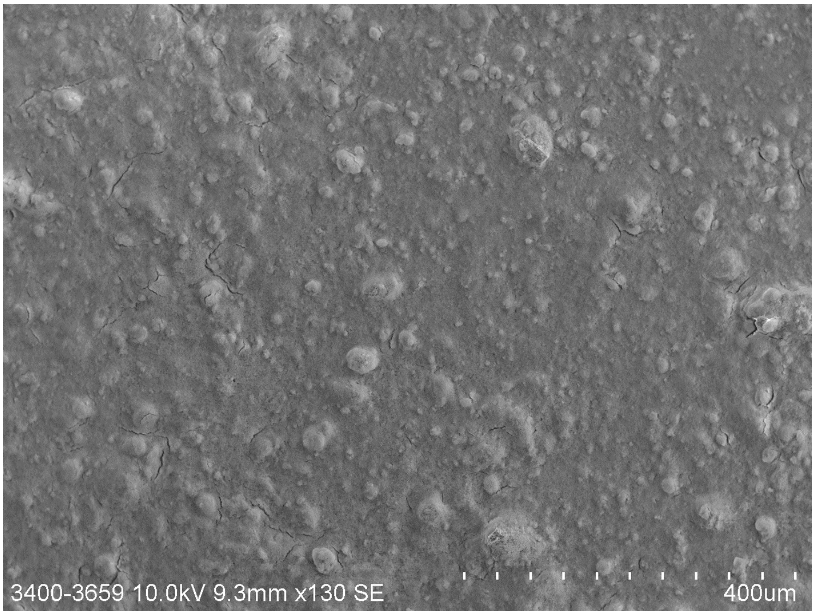

Supplementary Figure 29. Surface morphology of the microfluidic detection chip through SEM image.

Supplementary Table 1. Comparison between EPD and other common techniques, including EWOD [14,25-32], magnetic [33-44], acoustic [45-61], and thermal [62-68] based droplet actuation platform.

		EPD (this work)	EWOD		Magnetic	Acoustic	Thermal
			Air-based	Oil-based			
Generality with operable liquids	Droplet size	High (over 500 nL-1 mL, Fig. 3B)	Low (depends on electrode size, nL-10 µL) [25-27]		Medium (1 µL-200 µL) [33,34]	Medium (200 nL-50 µL) [45-47]	Low (depends on microheater size, 3 nL-1 µL) [62-64]
	Liquid types	High (Fig 3)	Low (high conductive/ permittivity, Fig. S2)	Low (high conductive/ permittivity, Fig. S2)	Medium (prefer high surface tension & low viscosity) [33,34]	High [47,48]	Medium (non-volatile liquid) [62]
	Other requirement	No	No	Immiscible with silicone oil [28]	Not catalyzed/reactive with magnetic nanoparticles [35,36]	No acids or bases on ZnO substrate [46]	Prefer liquid with highly temperature-dependent surface tension [62]
Compatibility with bio-samples	Body fluids	High (Fig.1D)	Low (Fig.S3)	Low (Fig.S3, S8)	High [37,38]	High [49,50]	Medium (heating may affect activity of biomarker inside) [65]
	Protein	High (Fig.4C)	Low (Fig. S9)	Medium (Fig. S10)	High [39]	Medium (acoustic heating may affect activity) [46,51,52]	Low (direct heating may damage activity) [66]
	Living cells	High (Fig.4D,E)	Low (Fig. 4E)	Low (Fig. S11)	High [40]	Medium (increased amplitude/ decreased frequency may break cell membranes) [53-56]	Low (direct heating may damage activity) [66]
	Bioassay process	High (Fig. 6)	Low (biofouling) [28]	High	Low (particle's peroxidase-like activity & non-transparency) [35,36,41]	Low (acoustic heating affects temperature-dependent detection) [46,52]	Low (cross-contamination) [64]
Compatibility with surroundings /substrates	Droplet surrounding medium	High (oil/air/oil-air interface, Fig. 4)	High (air/oil)		High (oil/air) [34]	High (air/oil) [45,46]	Low (air only) [62,64]
	Substrate surface	Medium (hydrophobic/ oil substrate, Fig. 4)	Medium (hydrophobic) [29]		Medium (hydrophobic) [34]	High (hydrophobic/ hydrophilic) [57]	Low (hydrophilic surface with hydrophobic pattern) [62,64]
	Substrate material	High (no demand)	Low (limited by thickness and permittivity) [30,31]		Medium (some require flexible substrate impregnated with magnetic materials) [42,43]	Low (piezoelectric materials) [46,58]	Medium (thickness and thermal properties of the substrate affect heating efficiency) [64]
Speed		High (22-60 mm/s, Fig. 3B)	High (140 mm/s) [14]	High (45 mm/s) [14]	High (3-50 mm/s) [33,37,42]	High (10-50 mm/s) [59]	Low (0.01-0.3 mm/s) [62,67]
Resolution		Depends on coil size, e.g., 1.5 mm (Fig. S21)	Depends on electrode size, e.g., 2 mm (Fig. S21)		Depends on electromagnet size, e.g., 1.9 mm (Fig. S21)	Depends on droplet volume and RF pulse duration, e.g., 1mm [57]	Depends on microheater size, e.g., 1mm [62,64]
Simplicity of programming		High (switch matrix, Fig. 5)	High (switch matrix) [32]		High (switch matrix/translation stage/alternating electromagnetic field) [34,44]	Low (modulate frequency, amplitude, and phase difference of one/multiple actuators) [60,61]	Medium (switch matrix, with an optional temperature feedback control) [64,68]
Working Voltage		Low (5.5V)	High (60 V-150 V, depends on liquid types, Fig. S2, S3, S8)		Medium (several-tens of volts) [33,34,37,44]	Medium (40 V-70 V) [46]	Medium (2.5V-25V) [62,64,67]
Cost of fabrication		Low (consumables: lower than US$1; control system: ~US$100)	Medium (consumables: US$42.6; control system: ~US$500)		Medium (consumables: lower than US$1; control system: ~US$100-US$1000) [33,34,37,44]	High (consumables: ~US$70; control system: ~US$600-US$3000) [60,61]	Medium (consumables: ~US$10; control system: ~US$100-US$1000) [62,64,68]

Reference for Supplementary Information

1 Pethig, R. Review Article—Dielectrophoresis: Status of the theory, technology, and applications. *Biomicrofluidics* **4**, doi:10.1063/1.3456626 (2010).

2 Çetin, B. & Li, D. Dielectrophoresis in microfluidics technology. *ELECTROPHORESIS* **32**, 2410-2427, doi:https://doi.org/10.1002/elps.201100167 (2011).

3 Renaudot, R., Agache, V., Daunay, B., Lambert, P., Kumemura, M., Fouillet, Y., Collard, D. & Fujita, H. Optimization of Liquid DiElectroPhoresis (LDEP) Digital Microfluidic Transduction for Biomedical Applications. *Micromachines* **2**, 258-273 (2011).

4 Fan, S.-K., Hsieh, T.-H. & Lin, D.-Y. General digital microfluidic platform manipulating dielectric and conductive droplets by dielectrophoresis and electrowetting. *Lab on a Chip* **9**, 1236-1242, doi:10.1039/B816535A (2009).

5 Velev, O. D., Prevo, B. G. & Bhatt, K. H. On-chip manipulation of free droplets. *Nature* **426**, 515-516, doi:10.1038/426515a (2003).

6 Zhao, K. & Li, D. Direct current dielectrophoretic manipulation of the ionic liquid droplets in water. *Journal of Chromatography A* **1558**, 96-106, doi:https://doi.org/10.1016/j.chroma.2018.05.020 (2018).

7 Jones, T. B., Fowler, J. D., Chang, Y. S. & Kim, C.-J. Frequency-Based Relationship of Electrowetting and Dielectrophoretic Liquid Microactuation. *Langmuir* **19**, 7646-7651, doi:10.1021/la0347511 (2003).

8 Wu, J., Li, X., Lin, T., Zhuang, L., Tang, B., Liu, F. & Zhou, G. Electric-Field-Induced Selective Directed Transport of Diverse Droplets. *ACS Applied Materials & Interfaces* **16**, 4126-4137, doi:10.1021/acsami.3c13792 (2024).

9 Chen, K., Quan, Y., Song, C., Xiang, N., Jiang, D., Sun, D., Yang, J., Yi, H. & Ni, Z. Accurate control of individual metallic nanowires by light-induced dielectrophoresis: Size-based separation and array-spacing regulation. *Sensors and Actuators A: Physical* **225**, 139-147, doi:https://doi.org/10.1016/j.sna.2014.07.025 (2015).

10 Gascoyne, P. R. & Vykoukal, J. Particle separation by dielectrophoresis. *Electrophoresis* **23**, 1973-1983, doi:10.1002/1522-2683(200207)23:13<1973::Aid-elps1973>3.0.Co;2-1 (2002).

11 Ahn, K., Kerbage, C., Hunt, T. P., Westervelt, R. M., Link, D. R. & Weitz, D. A. Dielectrophoretic manipulation of drops for high-speed microfluidic sorting devices. *Applied Physics Letters* **88**, doi:10.1063/1.2164911 (2006).

12 Xu, H., Clarke, A., Rothstein, J. P. & Poole, R. J. Viscoelastic drops moving on hydrophilic and superhydrophobic surfaces. *Journal of Colloid and Interface Science* **513**, 53-61, doi:https://doi.org/10.1016/j.jcis.2017.10.105 (2018).

13 Dash, S. & Garimella, S. V. Droplet Evaporation Dynamics on a Superhydrophobic Surface with Negligible Hysteresis. *Langmuir* **29**, 10785-10795, doi:10.1021/la402784c (2013).

14 Brassard, D., Malic, L., Normandin, F., Tabrizian, M. & Veres, T. Water-oil core-shell droplets for electrowetting-based digital microfluidic devices. *Lab on a Chip* **8**, 1342-1349, doi:10.1039/B803827A (2008).

15 Torkkeli, A. *Droplet microfluidics on a planar surface*. (VTT Technical Research Centre of Finland, 2003).

16 Olin, P., Lindström, S. B., Pettersson, T. & Wågberg, L. Water Drop Friction on Superhydrophobic Surfaces. *Langmuir* **29**, 9079-9089, doi:10.1021/la401152b (2013).

17 Vinay, T. V. & Varanakkottu, S. N. Separation of Floating Oil Drops Based on Drop-Liquid Substrate Interfacial Tension. *Langmuir* **35**, 10596-10600, doi:10.1021/acs.langmuir.9b01829 (2019).
18 Kralchevsky, P. A. & Nagayama, K. Capillary forces between colloidal particles. *Langmuir* **10**, 23-36 (1994).
19 Nagayama, K. & Kralchevsky, P. A. Universal Two-dimensional forces that Act on Particles at interfaces. *Current Opinion in Colloid & Interface Science* **59**, 101578, doi:https://doi.org/10.1016/j.cocis.2022.101578 (2022).
20 Bansal, S. & Sen, P. Effect of electrowetting induced capillary oscillations on coalescence of compound droplets. *Journal of Colloid and Interface Science* **530**, 223-232, doi:https://doi.org/10.1016/j.jcis.2018.05.090 (2018).
21 Wang, T., Andersen, S. I. & Shapiro, A. Coalescence of oil droplets in microchannels under brine flow. *Colloids and Surfaces A: Physicochemical and Engineering Aspects* **598**, 124864, doi:https://doi.org/10.1016/j.colsurfa.2020.124864 (2020).
22 Aarts, D. G. A. L., Lekkerkerker, H. N. W., Guo, H., Wegdam, G. H. & Bonn, D. Hydrodynamics of Droplet Coalescence. *Physical Review Letters* **95**, 164503, doi:10.1103/PhysRevLett.95.164503 (2005).
23 Pitois, O., Moucheront, P. & Chateau, X. Liquid Bridge between Two Moving Spheres: An Experimental Study of Viscosity Effects. *Journal of Colloid and Interface Science* **231**, 26-31, doi:https://doi.org/10.1006/jcis.2000.7096 (2000).
24 Li, W., Tang, X. & Wang, L. Photopyroelectric microfluidics. *Science Advances* **6**, eabc1693, doi:doi:10.1126/sciadv.abc1693 (2020).
25 Chen, J., Yu, Y., Li, J., Lai, Y. & Zhou, J. Size-variable droplet actuation by interdigitated electrowetting electrode. *Applied Physics Letters* **101**, doi:10.1063/1.4769433 (2012).
26 Gong, J. & Kim, C.-J. C. All-electronic droplet generation on-chip with real-time feedback control for EWOD digital microfluidics. *Lab on a Chip* **8**, 898-906, doi:10.1039/B717417A (2008).
27 Sung Kwon, C., Hyejin, M. & Chang-Jin, K. Creating, transporting, cutting, and merging liquid droplets by electrowetting-based actuation for digital microfluidic circuits. *Journal of Microelectromechanical Systems* **12**, 70-80, doi:10.1109/JMEMS.2002.807467 (2003).
28 Fan, S.-K., Hsu, Y.-W. & Chen, C.-H. Encapsulated droplets with metered and removable oil shells by electrowetting and dielectrophoresis. *Lab on a Chip* **11**, 2500-2508, doi:10.1039/C1LC20142E (2011).
29 Latip, E. A., Coudron, L., McDonnell, M., Johnston, I., McCluskey, D., Day, R. & Tracey, M. Protein droplet actuation on superhydrophobic surfaces: a new approach toward anti-biofouling electrowetting systems. *RSC advances* **7**, 49633-49648 (2017).
30 Chae, J. B., Kwon, J. O., Yang, J. S., Kim, D., Rhee, K. & Chung, S. K. Optimum thickness of hydrophobic layer for operating voltage reduction in EWOD systems. *Sensors and Actuators A: Physical* **215**, 8-16, doi:https://doi.org/10.1016/j.sna.2013.11.001 (2014).
31 Liu, H., Dharmatilleke, S., Maurya, D. K. & Tay, A. A. Dielectric materials for electrowetting-on-dielectric actuation. *Microsystem technologies* **16**, 449-460 (2010).
32 Shen, H.-H., Fan, S.-K., Kim, C.-J. & Yao, D.-J. EWOD microfluidic systems for biomedical applications. *Microfluidics and Nanofluidics* **16**, 965-987, doi:10.1007/s10404-014-1386-y (2014).
33 Long, Z., Shetty, A. M., Solomon, M. J. & Larson, R. G. Fundamentals of magnet-actuated droplet manipulation on an open hydrophobic surface. *Lab on a Chip* **9**, 1567-1575, doi:10.1039/B819818G (2009).

34 Zhang, Y. & Nguyen, N.-T. Magnetic digital microfluidics – a review. *Lab on a Chip* **17**, 994-1008, doi:10.1039/C7LC00025A (2017).
35 Gao, L., Zhuang, J., Nie, L., Zhang, J., Zhang, Y., Gu, N., Wang, T., Feng, J., Yang, D. & Perrett, S. Intrinsic peroxidase-like activity of ferromagnetic nanoparticles. *Nature nanotechnology* **2**, 577-583 (2007).
36 Wei, H. & Wang, E. Fe3O4 magnetic nanoparticles as peroxidase mimetics and their applications in H2O2 and glucose detection. *Analytical chemistry* **80**, 2250-2254 (2008).
37 Lin, H., Yu, W., A. Sabet, K., Bogumil, M., Zhao, Y., Hambalek, J., Lin, S., Chandrasekaran, S., Garner, O., Di Carlo, D. & Emaminejad, S. Ferrobotic swarms enable accessible and adaptable automated viral testing. *Nature* **611**, 570-577, doi:10.1038/s41586-022-05408-3 (2022).
38 Yu, W., Lin, H., Wang, Y., He, X., Chen, N., Sun, K., Lo, D., Cheng, B., Yeung, C., Tan, J., Di Carlo, D. & Emaminejad, S. A ferrobotic system for automated microfluidic logistics. *Science Robotics* **5**, eaba4411, doi:doi:10.1126/scirobotics.aba4411 (2020).
39 Tekin, H. C. & Gijs, M. A. M. Ultrasensitive protein detection: a case for microfluidic magnetic bead-based assays. *Lab on a Chip* **13**, 4711-4739, doi:10.1039/C3LC50477H (2013).
40 Yaman, S., Anil-Inevi, M., Ozcivici, E. & Tekin, H. C. Magnetic Force-Based Microfluidic Techniques for Cellular and Tissue Bioengineering. *Frontiers in Bioengineering and Biotechnology* **6**, doi:10.3389/fbioe.2018.00192 (2018).
41 Bajwa, I. U. & Sigaud, S. Effect of cell isolation magnetic particles on DNA quantification by UV absorbance spectrophotometry. *medRxiv*, 2023.2004. 2024.23288526 (2023).
42 Seo, K. S., Wi, R., Im, S. G. & Kim, D. H. A superhydrophobic magnetic elastomer actuator for droplet motion control. *Polymers for Advanced Technologies* **24**, 1075-1080, doi:https://doi.org/10.1002/pat.3190 (2013).
43 Zhou, Q., Ristenpart, W. D. & Stroeve, P. Magnetically Induced Decrease in Droplet Contact Angle on Nanostructured Surfaces. *Langmuir* **27**, 11747-11751, doi:10.1021/la2024633 (2011).
44 Cao, Q., Han, X. & Li, L. Configurations and control of magnetic fields for manipulating magnetic particles in microfluidic applications: magnet systems and manipulation mechanisms. *Lab on a Chip* **14**, 2762-2777, doi:10.1039/C4LC00367E (2014).
45 Guttenberg, Z., Müller, H., Habermüller, H., Geisbauer, A., Pipper, J., Felbel, J., Kielpinski, M., Scriba, J. & Wixforth, A. Planar chip device for PCR and hybridization with surface acoustic wave pump. *Lab on a Chip* **5**, 308-317, doi:10.1039/B412712A (2005).
46 Du, X. Y., Fu, Y. Q., Luo, J. K., Flewitt, A. J. & Milne, W. I. Microfluidic pumps employing surface acoustic waves generated in ZnO thin films. *Journal of Applied Physics* **105**, doi:10.1063/1.3068326 (2009).
47 Wang, Z. & Zhe, J. Recent advances in particle and droplet manipulation for lab-on-a-chip devices based on surface acoustic waves. *Lab on a Chip* **11**, 1280-1285, doi:10.1039/C0LC00527D (2011).
48 Bourquin, Y., Reboud, J., Wilson, R. & Cooper, J. M. Tuneable surface acoustic waves for fluid and particle manipulations on disposable chips. *Lab on a Chip* **10**, 1898-1901, doi:10.1039/C004506C (2010).
49 Chen, X., Zhang, C., Liu, X., Dong, Y., Meng, H., Qin, X., Jiang, Z. & Wei, X. Low-noise fluorescent detection of cardiac troponin I in human serum based on surface acoustic wave separation. *Microsystems & Nanoengineering* **9**, 141, doi:10.1038/s41378-023-00600-5 (2023).
50 Liu, X., Chen, X., Yang, Z., Xia, H., Zhang, C. & Wei, X. Surface acoustic wave based microfluidic devices for biological applications. *Sensors & Diagnostics* **2**, 507-528 (2023).

51 Girardo, S., Cecchini, M., Beltram, F., Cingolani, R. & Pisignano, D. Polydimethylsiloxane–LiNbO3 surface acoustic wave micropump devices for fluid control into microchannels. *Lab on a Chip* **8**, 1557-1563, doi:10.1039/B803967D (2008).
52 Renaudin, A., Chabot, V., Grondin, E., Aimez, V. & Charette, P. G. Integrated active mixing and biosensing using surface acoustic waves (SAW) and surface plasmon resonance (SPR) on a common substrate. *Lab on a Chip* **10**, 111-115, doi:10.1039/B911953A (2010).
53 Mutafopulos, K., Lu, P. J., Garry, R., Spink, P. & Weitz, D. A. Selective cell encapsulation, lysis, pico-injection and size-controlled droplet generation using traveling surface acoustic waves in a microfluidic device. *Lab on a Chip* **20**, 3914-3921 (2020).
54 Salehi-Reyhani, A., Gesellchen, F., Mampallil, D., Wilson, R., Reboud, J., Ces, O., Willison, K. R., Cooper, J. M. & Klug, D. R. Chemical-free lysis and fractionation of cells by use of surface acoustic waves for sensitive protein assays. *Analytical chemistry* **87**, 2161-2169 (2015).
55 Li, H., Friend, J., Yeo, L., Dasvarma, A. & Traianedes, K. Effect of surface acoustic waves on the viability, proliferation and differentiation of primary osteoblast-like cells. *Biomicrofluidics* **3**, doi:10.1063/1.3194282 (2009).
56 Yeo, L. Y. & Friend, J. R. Ultrafast microfluidics using surface acoustic waves. *Biomicrofluidics* **3**, doi:10.1063/1.3056040 (2009).
57 Renaudin, A., Tabourier, P., Zhang, V., Camart, J. C. & Druon, C. SAW nanopump for handling droplets in view of biological applications. *Sensors and Actuators B: Chemical* **113**, 389-397, doi:https://doi.org/10.1016/j.snb.2005.03.100 (2006).
58 Cecchini, M., Girardo, S., Pisignano, D., Cingolani, R. & Beltram, F. Acoustic-counterflow microfluidics by surface acoustic waves. *Applied Physics Letters* **92**, doi:10.1063/1.2889951 (2008).
59 Ai, Y. & Marrone, B. L. Droplet translocation by focused surface acoustic waves. *Microfluidics and Nanofluidics* **13**, 715-722, doi:10.1007/s10404-012-0990-y (2012).
60 Ding, X., Lin, S.-C. S., Kiraly, B., Yue, H., Li, S., Chiang, I.-K., Shi, J., Benkovic, S. J. & Huang, T. J. On-chip manipulation of single microparticles, cells, and organisms using surface acoustic waves. *Proceedings of the National Academy of Sciences* **109**, 11105-11109, doi:doi:10.1073/pnas.1209288109 (2012).
61 Zhou, Q., Sariola, V., Latifi, K. & Liimatainen, V. Controlling the motion of multiple objects on a Chladni plate. *Nature Communications* **7**, 12764, doi:10.1038/ncomms12764 (2016).
62 Darhuber, A. A., Valentino, J. P. & Troian, S. M. Planar digital nanoliter dispensing system based on thermocapillary actuation. *Lab on a Chip* **10**, 1061-1071, doi:10.1039/B921759B (2010).
63 Nguyen, N.-T. & Huang, X. Thermocapillary Effect of a Liquid Plug in Transient Temperature Fields. *Japanese Journal of Applied Physics* **44**, 1139, doi:10.1143/JJAP.44.1139 (2005).
64 Darhuber, A. A., Valentino, J. P., Troian, S. M. & Wagner, S. Thermocapillary actuation of droplets on chemically patterned surfaces by programmable microheater arrays. *Journal of Microelectromechanical Systems* **12**, 873-879, doi:10.1109/JMEMS.2003.820267 (2003).
65 Meng, J., Li, S., Li, J., Yu, C., Wei, C. & Dai, S. AC electrothermal mixing for high conductive biofluids by arc-electrodes. *Journal of Micromechanics and Microengineering* **28**, 065004, doi:10.1088/1361-6439/aab39b (2018).
66 Won, B. J., Lee, W. & Song, S. Estimation of the thermocapillary force and its applications to precise droplet control on a microfluidic chip. *Sci Rep* **7**, 3062, doi:10.1038/s41598-017-03028-w (2017).
67 Sammarco, T. S. & Burns, M. A. Thermocapillary pumping of discrete drops in microfabricated analysis devices. *AIChE Journal* **45**, 350-366, doi:https://doi.org/10.1002/aic.690450215 (1999).

68 Liu, M.-C., Wu, J.-G., Tsai, M.-F., Yu, W.-S., Lin, P.-C., Chiu, I. C., Chin, H.-A., Cheng, I. C., Tung, Y.-C. & Chen, J.-Z. Two dimensional thermoelectric platforms for thermocapillary droplet actuation. *RSC Advances* **2**, 1639-1642, doi:10.1039/C1RA00896J (2012).
