# Supplementary material for: A droplet robotic system enabled by electret-induced polarization on droplet
Source: Nat Commun. 2024 Jul 23;15:6220. doi: 10.1038/s41467-024-50520-9 (PMC11266649; doi:10.1038/s41467-024-50520-9)
Supplement: Supplementary file 3 — Description of Additional Supplementary Files [file 41467_2024_50520_MOESM3_ESM.pdf]

Description of Additional Supplementary Files

File Name: Supplementary Movie 1
Description: Droplet actuated by EPD-based droplet robotic system with different trajectories.

File Name: Supplementary Movie 2
Description: Comparison of the actuation of various inorganic/organic liquids on EWOD and EPD-based droplet robotic system.

File Name: Supplementary Movie 3
Description: Comparison of the actuation of various bio-fluids on EWOD and EPD-based droplet robotic system.

File Name: Supplementary Movie 4
Description: Actuation of protein solution with EWOD.

File Name: Supplementary Movie 5
Description: Actuation of protein solution with EPD.

File Name: Supplementary Movie 6
Description: Simulated Maxwell stress tensor applied on droplet by slice-shape electret and gripper-shape electret.

File Name: Supplementary Movie 7
Description: Multiple microfluidic functions of the EPD-based droplet robotic system.

File Name: Supplementary Movie 8
Description: Step-by-step demonstration of the workflow of the EPD-based droplet robotic system for automated lithium detection.
