# Supplementary material for: A droplet robotic system enabled by electret-induced polarization on droplet
Source: Nat Commun. 2024 Jul 23;15:6220. doi: 10.1038/s41467-024-50520-9 (PMC11266649; doi:10.1038/s41467-024-50520-9)
Supplement: Supplementary file 12 — Reporting Summary [file 41467_2024_50520_MOESM12_ESM.pdf]

nature portfolio

Corresponding author(s): Ho Cheung Shum & Haisong Lin

Last updated by author(s): Jul 6, 2024

Reporting Summary

Nature Portfolio wishes to improve the reproducibility of the work that we publish. This form provides structure for consistency and transparency in reporting. For further information on Nature Portfolio policies, see our Editorial Policies and the Editorial Policy Checklist.

Statistics

For all statistical analyses, confirm that the following items are present in the figure legend, table legend, main text, or Methods section.

n/a	Confirmed	
	☒	The exact sample size (*n*) for each experimental group/condition, given as a discrete number and unit of measurement
	☒	A statement on whether measurements were taken from distinct samples or whether the same sample was measured repeatedly
	☒	The statistical test(s) used AND whether they are one- or two-sided<br>*Only common tests should be described solely by name; describe more complex techniques in the Methods section.*
☒		A description of all covariates tested
☒		A description of any assumptions or corrections, such as tests of normality and adjustment for multiple comparisons
	☒	A full description of the statistical parameters including central tendency (e.g. means) or other basic estimates (e.g. regression coefficient) AND variation (e.g. standard deviation) or associated estimates of uncertainty (e.g. confidence intervals)
	☒	For null hypothesis testing, the test statistic (e.g. *F*, *t*, *r*) with confidence intervals, effect sizes, degrees of freedom and *P* value noted<br>*Give P values as exact values whenever suitable.*
☒		For Bayesian analysis, information on the choice of priors and Markov chain Monte Carlo settings
☒		For hierarchical and complex designs, identification of the appropriate level for tests and full reporting of outcomes
☒		Estimates of effect sizes (e.g. Cohen's *d*, Pearson's *r*), indicating how they were calculated

Our web collection on statistics for biologists contains articles on many of the points above.

Software and code

Policy information about availability of computer code

Data collection	ImageJ 1.51v Java 1.8.0_112 for collecting fluorescence intensity data, Tracker 6.0.10 for collecting data of droplet movement, Auto Curve Analysis Tracer Software for collecting data from Keithley 6514, Arduino IDE 1.8.19 for programming the control matrix
Data analysis	Origin 2018 and Microsoft Excel for analyzing and plotting data, ImageJ 1.51v Java 1.8.0_112 for analyzing microscopic image.

For manuscripts utilizing custom algorithms or software that are central to the research but not yet described in published literature, software must be made available to editors and reviewers. We strongly encourage code deposition in a community repository (e.g. GitHub). See the Nature Portfolio guidelines for submitting code & software for further information.

Data

Policy information about availability of data

All manuscripts must include a data availability statement. This statement should provide the following information, where applicable:

- Accession codes, unique identifiers, or web links for publicly available datasets
- A description of any restrictions on data availability
- For clinical datasets or third party data, please ensure that the statement adheres to our policy

Data generated in this study are provided in the paper and/or the Supplementary Information. Source data are provided with this paper.

Research involving human participants, their data, or biological material

Policy information about studies with human participants or human data. See also policy information about sex, gender (identity/presentation), and sexual orientation and race, ethnicity and racism.

Reporting on sex and gender	The information of sex and gender were not required for this study.
Reporting on race, ethnicity, or other socially relevant groupings	The information of race, ethnicity, or other socially relevant groupings were not required for this study.
Population characteristics	The information of population characteristics were not required for this study.
Recruitment	Human urine and saliva samples were obtained following The University of Hong Kong, Human Research Ethics Committee approved research project, HREC No. EA230092 with consent from all participants. Participants were recruited as volunteers. There was no perceived bias in the recruitment process.
Ethics oversight	Human Research Ethics Committee of The University of Hong Kong

Note that full information on the approval of the study protocol must also be provided in the manuscript.

Field-specific reporting

Please select the one below that is the best fit for your research. If you are not sure, read the appropriate sections before making your selection.

☒ Life sciences ☐ Behavioural & social sciences ☐ Ecological, evolutionary & environmental sciences

For a reference copy of the document with all sections, see nature.com/documents/nr-reporting-summary-flat.pdf

Life sciences study design

All studies must disclose on these points even when the disclosure is negative.

Sample size	Sample numbers are provided in the corresponding figure caption or methods. Sample size was determined to warrant at least three replicates of biologically independent experiments for sufficient demonstration and statistical analysis, as standard for biological experiments.
Data exclusions	No data were excluded from the analysis.
Replication	All experiments were successfully replicated for at least three times independently.
Randomization	All samples were allocated into experimental groups randomly.
Blinding	The investigators were blinded to the group allocation during data collection and/or analysis.

Reporting for specific materials, systems and methods

We require information from authors about some types of materials, experimental systems and methods used in many studies. Here, indicate whether each material, system or method listed is relevant to your study. If you are not sure if a list item applies to your research, read the appropriate section before selecting a response.

Materials & experimental systems

n/a	Involved in the study	
☒	☐	Antibodies
☒	☐	Eukaryotic cell lines
☒	☐	Palaeontology and archaeology
☒	☐	Animals and other organisms
☒	☐	Clinical data
☒	☐	Dual use research of concern
☒	☐	Plants

Methods

n/a	Involved in the study	
☒	☐	ChIP-seq
☒	☐	Flow cytometry
☒	☐	MRI-based neuroimaging

Plants

Seed stocks	No plants were used in this study.
Novel plant genotypes	No plants were used in this study.
Authentication	No plants were used in this study.
